# Mean hydrography on the continental shelf from 26 repeat glider deployments along Southeastern Australia

**DOI:** 10.1038/sdata.2016.70

**Published:** 2016-08-30

**Authors:** Amandine Schaeffer, Moninya Roughan, Tim Austin, Jason D. Everett, David Griffin, Ben Hollings, Edward King, Alessandra Mantovanelli, Stuart Milburn, Benedicte Pasquer, Charitha Pattiaratchi, Robin Robertson, Dennis Stanley, Iain Suthers, Dana White

**Affiliations:** 1 UNSW Australia, Sydney 2052, Australia; 2 Sydney Institute of Marine Science, Sydney 2088, Australia; 3 CSIRO Oceans and Atmosphere, Hobart 7001, Australia; 4 Australian National Facility for Ocean Gliders, Perth 6009, Australia; 5 Australian Ocean Data Network, Hobart 7001, Australia; 6 University of Western Australia, Perth 6009, Australia

**Keywords:** Marine biology, Physical oceanography

## Abstract

Since 2008, 26 glider missions have been undertaken along the continental shelf of southeastern Australia. Typically these missions have spanned the continental shelf on the inshore edge of the East Australian Current from 29.5–33.5*°*S. This comprehensive dataset of over 33,600 CTD profiles from the surface to within 10 m of the bottom in water depths ranging 25–200 m provides new and unprecedented high resolution observations of the properties of the continental shelf waters adjacent to a western boundary current, straddling the region where it separates from the coast. The region is both physically and biologically significant, and is also in a hotspot of ocean warming. We present gridded mean fields for temperature, salinity and density, but also dissolved oxygen and chlorophyll-a fluorescence indicative of phytoplankton biomass. This data will be invaluable for understanding shelf stratification, circulation, biophysical and bio-geochemical interactions, as well as for the validation of high-resolution ocean models or serving as teaching material.

## Background & Summary

Coastal oceans are very dynamic areas, influenced by coastal waters as well as by the open ocean. In particular, western boundary currents such as the Gulf Stream and the East Australian Current (EAC) occasionally intrude onto the adjacent shelf and drive cross-shelf transport, strongly influencing the hydrography of the water masses. Furthermore, estuarine outflow from rainfall in continental shelf regions drives strong horizontal and vertical salinity gradients, while local upwelling-favorable wind stress results in cold nutrient-rich water uplift for the growth of patchy phytoplankton blooms. This range of forcings leads to submesoscale processes that require measurements at high spatial resolution (ref. 1).

Along the southeastern coast of Australia, the EAC and its eddy field dominates the meso-scale circulation. This has been well described in part due to an Australian climatology, the CSIRO Atlas of Regional Seas (CARS, refs 2,^3^). CARS provides a gridded atlas of the ocean water properties with a horizontal resolution of 0.5*°* (~50 km), with different versions depending on the type of input dataset used, namely the World Ocean Database 2005, WOCE Global Hydrographic Program, research vessel profiles and ARGO floats. However, this resolution as well as the smooth bathymetry and the paucity of nearshore observations prevents this product from resolving the hydrography and dynamics on the narrow shelves.

In parallel, the Integrated Marine Observing System (IMOS, refs 4-6, www.imos.org.au) has become a key component of the ocean observation strategy around Australia since 2008. IMOS aims to deliver sustainable observations, with mooring measurements being the dominant source of *in situ* data, resulting in a lack of spatial coverage. Ocean gliders provide better spatial coverage, at the expense of temporal coverage. However, since the beginning of regular glider deployments, the density of high-resolution *in situ* observations has increased sufficiently to provide a representative mean state of the shelf waters with high cross-shelf resolution.

This glider dataset has been successfully used to map the influence of the EAC, its separation and the resulting uplift and upwelling on the southeastern Australian shelf^7^ based on the measurement of temperature, salinity and depth-averaged velocities. The characteristic length-scales of variability for biogeochemical and physical parameters have also been quantified from this dataset, confirming the importance of submesoscale processes, particularly across the shelf and for biologically-influenced processes^1^. Some of the early individual glider missions were the focus of process studies on eddy dynamics, highlighting shelf water entrainment^8^ and water mass transport within eddies^9^, as well as the flooding of a warm core eddy by the EAC^10^.

Here we present the high-resolution mean hydrography of the continental shelf along southeastern Australia, from repeat glider deployments over 8 years between 29.5*°*S and 33.5*°*S. The data were quality controlled, and gridded using the same method as ^7^, providing for the first time a mean state of the shelf temperature, salinity and density, but also Chlorophyll-a fluorescence (CHL) and dissolved oxygen (DO) concentrations.

## Methods

### Gliders

Ocean gliders are autonomous underwater vehicles (AUVs) that control their vertical excursions in the water column by changing their buoyancy relative to the surrounding water mass. The vertical momentum is then partially converted into horizontal motion using two lateral wings. Gliders navigate following pre-programmed waypoints that can be adjusted when they surface, update their GPS position and access satellite communications^11^, providing an invaluable tool for ocean *in situ* observations^12^.

### Glider deployments

Various glider deployment strategies were tested in this region. Initially gliders were deployed into western boundary current eddies^9,10^, which proved unsustainable due to retrieval difficulties when gliders were swept offshore. Subsequently by the end of 2010, a strategy was trialed whereby the glider only traversed the continental shelf region.

**Figure 1:**
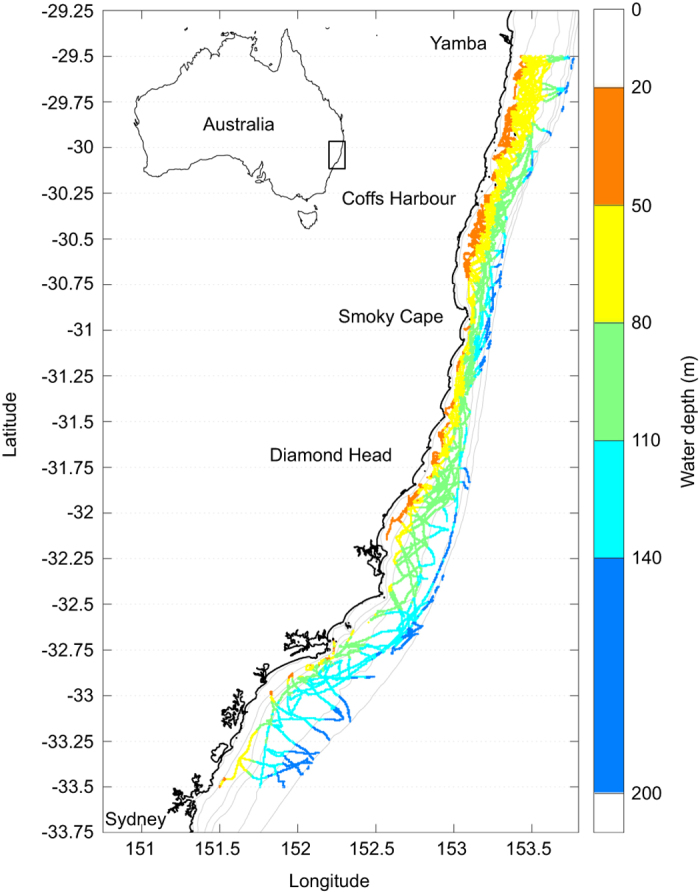
Study area and glider paths from the 26 shelf deployments between 2008 and 2015, adapted from (refs 7). The along-shelf grid used for the mean calculation is shown by horizontal lines (resolution of 0.25 *°*) while the discretization across the shelf follows the isobaths (20, 50, 80, 110, 140, 200 m) as indicated by the along-track colors.

As depth-averaged currents in the region often exceed 1 m s^−1^ and gliders have limited forward propulsion (<0.5 m s^−1^), repeat lines typical of other more quiescent regions were rarely successful. A zig-zag pattern was devised with the glider traversing the continental shelf, while being advected downstream with the dominant flow (Fig. 1).

**Figure 2:**
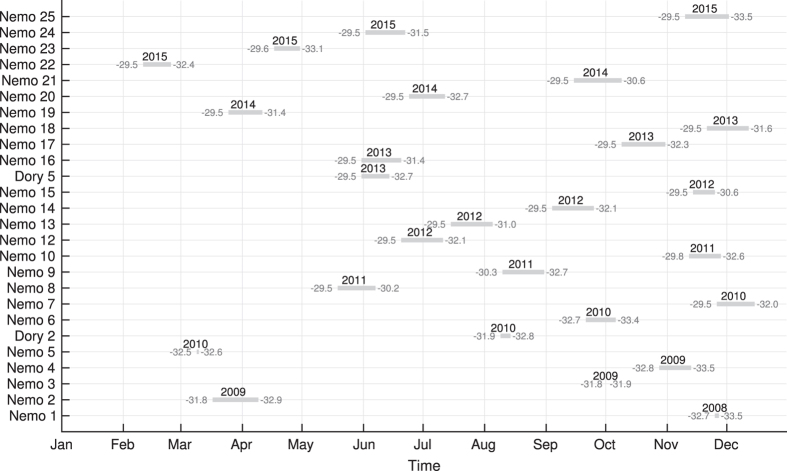
List of glider deployments per month (x-axis). Nemo and Dory refer to Slocum and Seaglider vehicles (mission names), respectively. Northernmost and Southernmost latitude reached during each deployment are indicated in gray at the ends of each line and the year is also shown (2008–2015). Gray lines show the length of the whole glider missions over the continental shelf (bathymetry <200 m).

**Figure 3:**
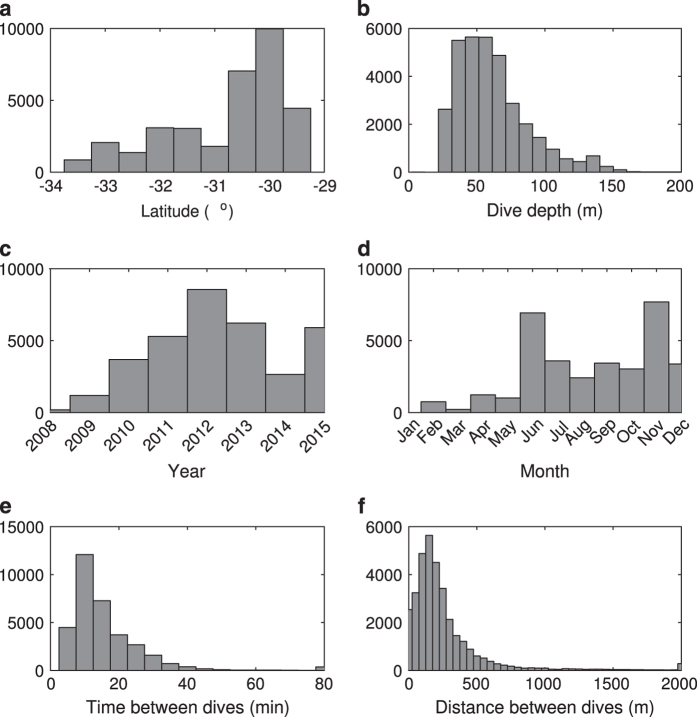
Distribution of glider profiles from the 26 missions by (**a**) latitude, (**b**) dive depth (25 m being the minimum depth used to define a cast), (**c**) year, (**d**) month, (**e**) time and (**f**) distance between dives.

The gliders were deployed seasonally, i.e., 3–4 missions per year (Fig. 2), dependent upon weather and other commitments. Deployment was typically from Yamba (16 out of 26 missions), NSW, 29.5*°*S in ~40 m of water (Fig. 1), leading to high profile density north of 31*°*S (Fig. 3a). The gliders were then sent to waypoints offshore (south-east), towards the 200 m isobath, and then back inshore (south-west), generating a zig-zag poleward track while being advected by the EAC (Fig. 1). Missions typically lasted around three weeks (Fig. 2, Table 1), as determined by battery life and a weather window for retrieval. During this time the gliders traversed up to 2–3 degrees of latitude, and undertook up to 2,866 dives on the shelf, generating as many high density CTD profiles (Table 1). In this work we focus on profiles obtained on the continental shelf (inshore of 200 m -isobath and between latitudes 29.5*°*S and 33.5*°*S). Individual mission tracks and profile data can be obtained at https://portal.aodn.org.au/ and browsed at http://oceancurrent.imos.org.au/gliders/index.php

The whole dataset consists of 26 glider missions between 2008 and 2015 (Figs 2 and 3c, Table 1) that provided a total of 33,600 profiles between the surface (or within 5 m) and 200 m. Dives that only penetrated from the surface to<25 m are not included in this dataset, leading to the highest density of dive depths between 30 and 70 m (Fig. 3b) and median maximum depths of 142 m (Table 1). Profiles were acquired throughout the year (Fig. 2), but with higher occurrence between June and December, i.e., the Austral winter to early summer (Fig. 3d).

### Measurements

The specific platforms used were mainly shallow Slocum gliders (8 G1 and 16 G2 instruments, standard buoyancy engine, codename ‘Nemo’), and 2 Seagliders (‘1kA’ model, standard fairing, codename ‘Dory’) that spanned the shelf for a few days before progressing offshore. They were typically instrumented with a Seabird-CTD (Conductivity—Temperature—Depth, pumped for Slocum gliders deployed after July 2011), an optical sensor (WETLabs) and an oxygen optode (Aanderaa). Details on the sensor models and calibration dates are available for each deployment in the file’s metadata. Amongst the parameters measured, we focus on temperature, salinity, density (calculated from the Thermodynamic Equation Of Seawater—2010, TEOS-10, (refs 13)), CHL (from fluorescence measurements, with excited/emitted wavelengths of 470/695 nm) and DO concentrations. Colored dissolved organic matter (CDOM) and optical backscatter are disregarded in this study due to insufficient quality control.

Each parameter is initially associated with a time and pressure (depth), while geographic coordinates are obtained during the glider surfacing via GPS and linearly interpolated during dives. Each vertical cast is defined from the deepest pressure measurement for each saw-tooth dive deeper than 25 m depth. The corresponding up—and down-casts are binned with 2 m vertical intervals for each horizontal cell. The time and distance between consecutive profiles on the shelf are short, usually 10–15 min and 100–200 m (Fig. 3e,f).

### Quality control and profile adjustments

All sensors are calibrated by the manufacturer every few missions. Calibration dates for CTD, bio-optical sensors and oxygen optodes are listed in the file’s metadata.

Major issues with CTD data are usually related to salinity calculations. In particular, the thermal lag effect (different time responses for temperature, pressure and conductivity) can lead to spurious salinity spikes, especially when using unpumped CTDs that do not allow for a constant flow inside the conductivity cell (used for missions before June 2011). Post-processing correction is routinely performed by IMOS using temperature alignment, thermal-lag correction and conductivity alignment, based on methods from^14^ and^15^. Details are available in http://content.aodn.org.au/Documents/IMOS/Facilities/Ocean_glider/SlocumCTD_data_corrections.pdf.

The quality of DO measurements can also be problematic. Before the deployments datasets are released, Oxygen concentrations from Aanderaa Optodes were recomputed following^15^ with phase and time lag correction, also using the CTD temperature rather than the optode’s sensor (see http://content.aodn.org.au/Documents/IMOS/Facilities/Ocean_glider/SlocumOptode_data_corrections.pdf for further details). Unfortunately, early missions (5 in 2008–2010) could not be corrected for phase issues due to missing parameters. In each of these cases, data were visually checked and judged to be of adequate quality. DO measurements for depths <1 m are disregarded as they might be biased by air-sea interaction.

**Figure 4:**
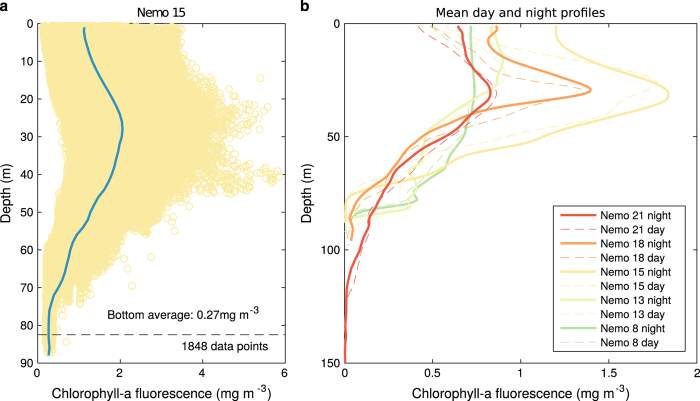
Post-processing quality control of CHL: (**a**) Example of offset estimation for the Nemo 15 mission, based on the average value of the deepest measurements. Circles show each measurement and the mean profile is overlaid in blue. The average bottom (5 m) values and the corresponding data point numbers are indicated. (**b**) Mean night and day CHL profiles for selected missions (see Table 1 for names) showing a surface underestimation due to quenching for day profiles. The quality control procedures are explained in section 2.4.

**Figure 5:**
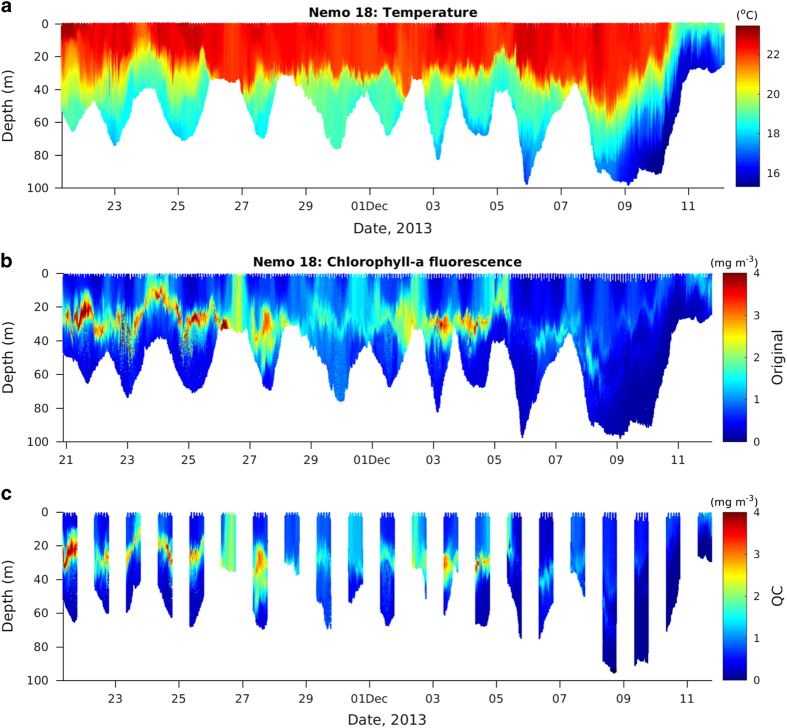
Example of *in situ* glider measurements for deployment Nemo 18: (**a**) temperature, (**b**) CHL before and (**c**) after additional QC (outlier removals, offset and night time only). The saw tooth pattern in depth indicates offshore and onshore movement of the glider along its track on the shelf.

As systematic bottle samples for calibration are lacking, post-processing was conducted to reduce measurement errors and homogenize the CHL dataset. For optical sensors, drift within missions were controlled by taking dark counts before and after deployments. Sensors and data were then checked for biofouling effects and suspicious measurements flagged before the release of individual data files through IMOS. For the purpose of this climatology, extra quality control was performed on the selected deployments. First, CHL outliers were removed from the time-series, based on the log-normal distribution of CHL and using a threshold of the mean+3 standard deviations. Second, due to the use of different sensors, CHL profiles were adjusted for each mission for cross sensor compatibility based on the assumption that CHL concentration should be nil at depths where light limits phytoplankton blooms. An offset value was estimated for each mission following^16^ and^17^ based on the average measurements over the 5 deepest meters (10 m for deep missions with dives >100 m), as shown in Fig. 4a. For this particular analysis, profiles offshore were considered when available, leading to maximum depths between 86 m and 1000 m (Table 1). The resulting offset value was then removed from all measurements of the deployment. We found offset values usually smaller than 0.1 mg m^−3^ with a maximum of 0.51 mg m^−3^ for the Nemo 23 mission (Table 1). Finally, in order to limit bias due to daytime quenching, only fluorescence measurements that were taken between sunset and sunrise were kept. Examples of the typical underestimation of fluorescence in the surface layers during daytime compared to nighttime is presented in Fig. 4c for a few glider missions. The overall improvement of the data quality is illustrated in Fig. 5b, showing the difference between raw CHL and QCed CHL for the Nemo 18 mission.

### Gridded mean

**Figure 6:**
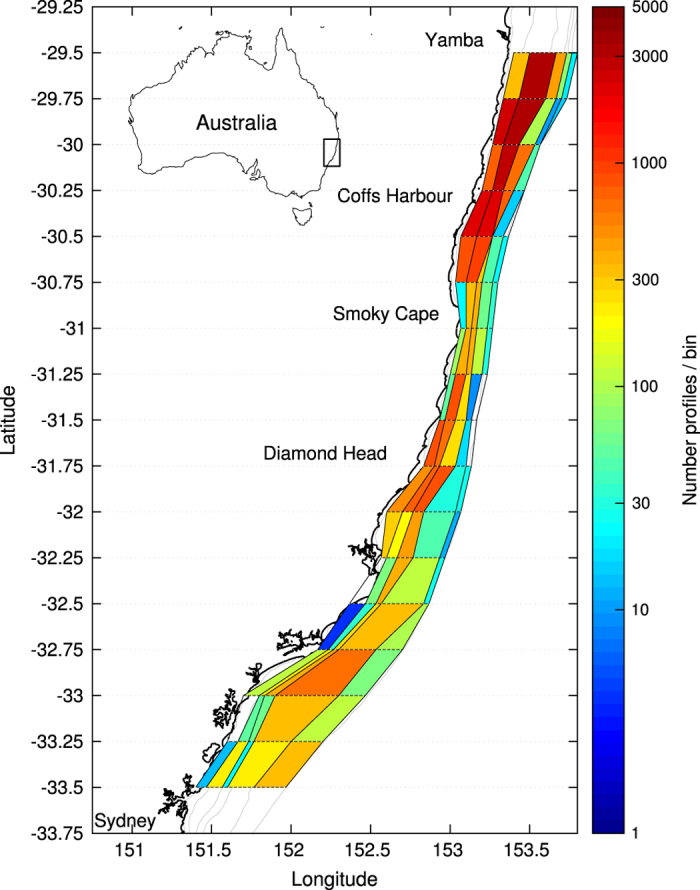
Binned total number of profiles from all glider missions. The along-shelf (resolution of 0.25 *°*) and across-shelf discretization based on isobaths (20, 50, 80, 110, 140 and 200 m) are shown.

The density of the observations is much higher across than along the shelf as a result of the shelf’s geometry, ocean dynamics and deployment strategy. In order to take this into account, a grid was chosen to be regular along latitudes, with a resolution of 0.25*°* (~25 km) but following the isobaths across the shelf as in (ref. 7), rather than the distance from the coastline. The resulting grid consists of 16 cells in latitude from 29.5*°*S to 33.5*°*S and 5 cells in longitude delimited by the 20, 50, 80, 110, 140 and 200 m isobaths, as shown in Figs 1 and 6.

Mean profiles (surface to 200 m) of temperature, salinity, density, CHL and DO were computed for each bin and each of the 26 glider missions. Table 1 presents information for each glider mission, such as the start and end date, the corresponding duration in days, number of profiles and maximum depth reached. Note that these data are relative to shelf observations only (inshore of the 200 m isobath), not taking into account potential offshore excursions of the glider. Table 1 also presents the mean and standard deviation of each measured parameter for the different missions.

**Figure 7:**
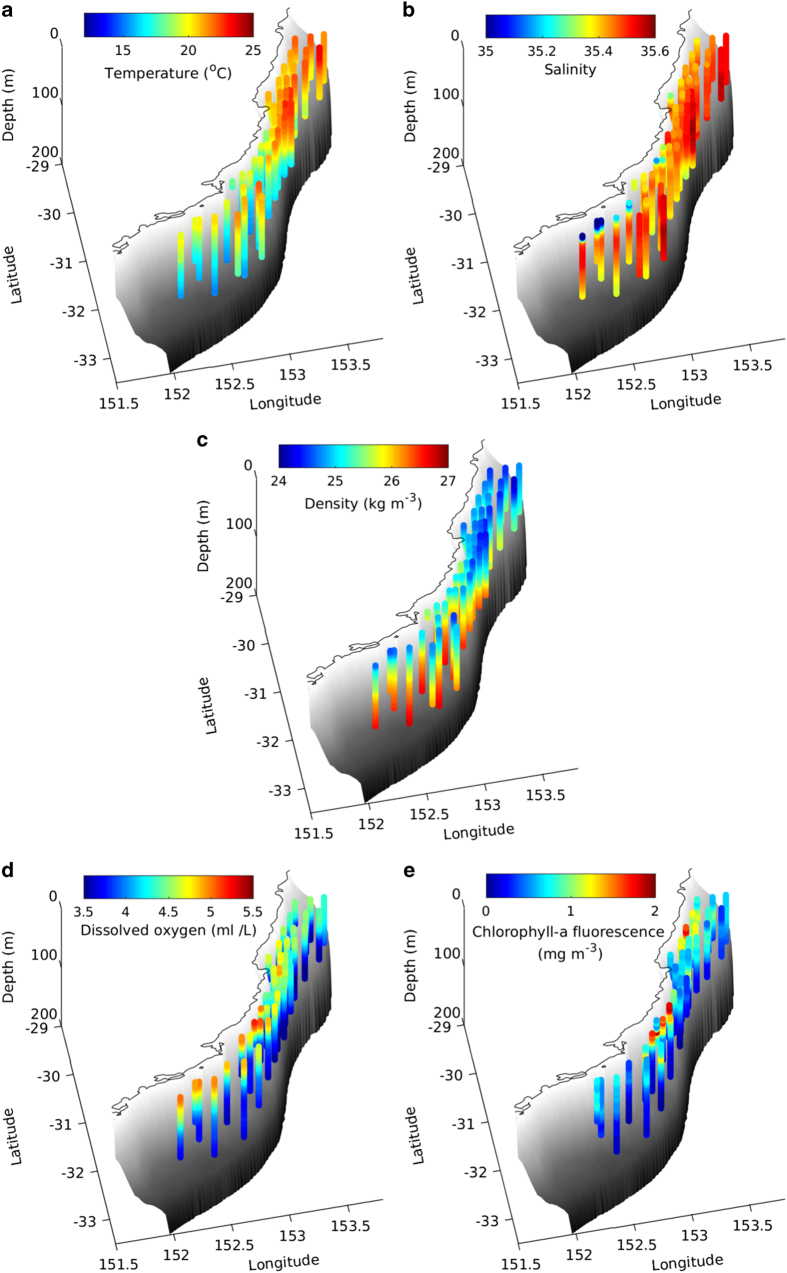
3D mean gridded profiles of temperature (**a**), salinity (**b**), density (**c**), DO (**d**) and CHL (**e**). The coastline between 29 and 33.5*°*S is indicated and grey shadings show the bathymetry from the surface (white) to 200 m depth (dark gray). Note that the fewer profiles for CHL is a consequence of more selective QC (especially night time limitation).

**Figure 8:**
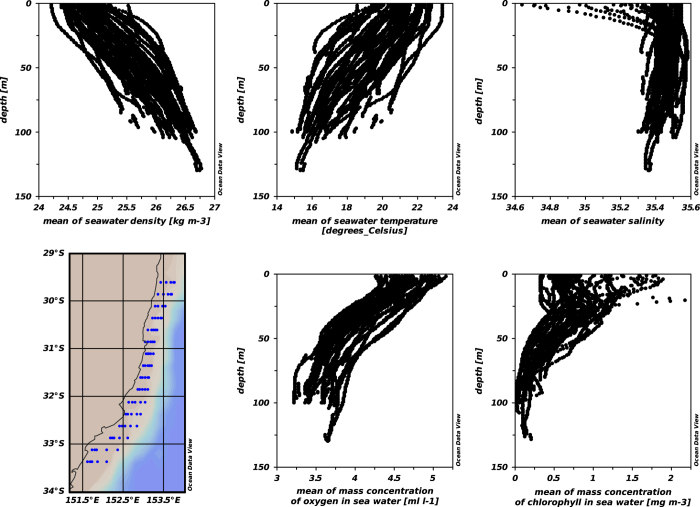
Visualization of the content of the data file ‘Mean_hydrography_NSWshelf_gliders_2008–2015.nc’ with Ocean Data View software (freely available, Schlitzer, R., Ocean Data View, odv.awi.de, 2015). Different panels show the gridded region with bathymetry shadings and profiles of density, temperature, salinity, DO and CHL for all cells.

Mean profiles were also computed from the averages over all glider missions, leading to a 3D average water mass characteristic over the shelf, as shown in Figs 7 and 8. The total number of profiles used to compute these mean values for each bin is shown in Fig. 6, ranging from 4 to 3310. Only bins where >30 profiles from at least 4 different missions are considered.

## Data Records

Data records are available at http://data.aodn.org.au/?prefix=UNSW/NSW_Glider_climatology/ (Data Citation 1) with two files, named ‘Mean_hydrography_NSWshelf_gliders_2008–2015.nc’ and ‘Mean_hydrography_NSWshelf_per_glider_2008–2015.nc’. The shelf water mass dataset is provided following IMOS NetCDF format and complies with the Climate and Forecasting (CF, version 1.6) conventions (http://content.aodn.org.au/Documents/IMOS/Conventions/IMOS_NetCDF_Conventions.pdf). The variables are detailed in Table 2. Each average profile is geolocated from its *I* and *J* bin numbers, associated to longitude and latitude coordinates. A third dimension is ‘Depth’ in meters, being positive downward from 1 to 200 m deep. All mean profiles are presented in Fig. 8 and the three-dimensional mean hydrography on the continental shelf is presented in Fig. 7 for each variable (temperature, salinity, density, CHL and DO).

While mean profiles over all glider deployments depended only on the latter three dimensions (latitude, longitude and depth), mean profiles per gliders also depend on a fourth dimension corresponding to the glider mission, number ‘GLIDER’, ranging from 1 to 26. The corresponding glider start and end times for each glider mission as well as the number of profiles each glider provided per bin (Table 1) are also specified in the data files.

A complete set of metadata is included with the variable description and units. As data were already quality controlled, following IMOS standards (only quality flags 1 were kept—‘good data’, http://content.aodn.org.au/Documents/IMOS/Facilities/Ocean_glider/glider_data_management.pdf) and the post-processing methods presented in section 2.4, no quality control flag is provided in the dataset files presented here. Non gridded data from each individual glider mission can be downloaded from the AODN data repository (https://portal.aodn.org.au/) as a NetCDF file. The corresponding IMOS file names for the missions used here are indicated in our metadata.

## Technical Validation

**Figure 9:**
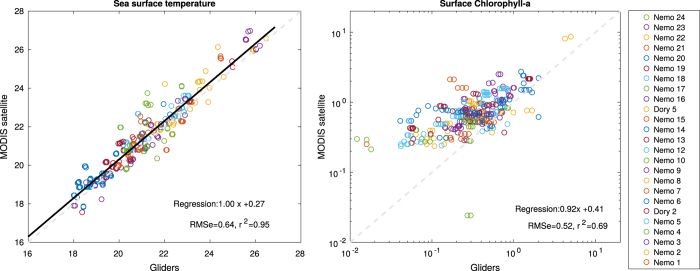
Surface (2 m depth) glider temperature (*°*C) and chlorophyll-a concentration (mg m^−3^, log scale) compared to MODIS satellite values for each match-up in time and space. Colors show glider missions described in Table 1. Linear regressions, root mean squared errors (RMSe) and correlation coefficients (r^2^) are specified. Statistics are carried on 414 and 373 data points, respectively, considering values <8 mg m^−3^ and >0.03 mg m^−3^ for chlorophyll-a.

Considering the large number and high resolution glider measurements, independent observations with matching times and locations are rare. Satellite ocean color was previously used by^17^ to produce a merged satellite and *in situ* fluorescence ocean chlorophyll product in the Mediterranean Sea. Here we present a comparison of surface temperature and CHL from gliders and satellite, using ocean color and sea surface temperature (SST) products from the Moderate Resolution Imaging Spectroradiometer (MODIS, http://oceancolor.gsfc.nasa.gov) sensor on the NASA Aqua spacecraft. Chlorophyll-a and temperature at 2 m below the surface from all glider missions were extracted together with their time stamp and coordinates. Level 2 (non-gridded) satellite observations were then matched if a satellite passed over the area within 25 min of the *in-situ* measurements, leading to a total of 414 matches. The nine nearest MODIS pixels were averaged (where valid) to provide the estimate of the satellite observation, which correspond to a mean distance of 4 km from the glider measurement. For the chlorophyll-a concentrations derived from the ocean color, the Garver-Siegel-Maritorena (GSM) product^18^ was chosen over the NASA standard OC3 product because of its capacity to better account for the optical complexity due to dissolved organic matter, which is more likely to be found in the shelf and near-shore environment. The MODIS longwave SST product was used for the temperature. The comparisons between the two datasets are presented in Fig. 9.

Surface temperatures from gliders and satellite are in very good agreement, with a linear regression slope of 1, correlation coefficient of *r*^2^=0.95 and root mean square error (RMSe) of 0.64*°C*^2^. Chlorophyll-a concentration is more challenging to match, due to measurement errors, calibration and unflagged outliers remaining in both datasets. The agreement is still good, with a slope of 0.92 and correlation coefficient of *r*^2^=0.69. However, the satellite (glider) measurements tend to over-estimate (under-estimate), especially for low Chlorophyll-a concentrations (<1 mg m^−3^).

## Usage Notes

This dataset can be used for example to extract typical seawater properties in the region, investigate spatial variability or validate model outputs. While all data included are quality controlled, it is to the user’s discretion to determine the significance of the means based on the number of data points provided for each bin. Any use of this data must be acknowledged using the following statement: ‘*This data was sourced from the Integrated Marine Observing System (IMOS)*—*IMOS is a national collaborative research infrastructure, supported by the Australian Government.*’

The dataset presented here is contributing to our understanding of shelf spatial variability in a critical zone where large scale circulation (EAC) and coastal processes meet. The high cross-shelf and vertical resolutions are unique features that were only possible from repeat glider observations. In addition, glider data include physical and biogeochemical measurements, thereby contributing to reducing the gap between complementary disciplines in ocean sciences.

## Additional Information

**How to cite this article:** Schaeffer, A. *et al.* Mean hydrography on the continental shelf from 26 repeat glider deployments along Southeastern Australia. *Sci. Data* 3:160070 doi: 10.1038/sdata.2016.70 (2016).

## Supplementary Material



## Figures and Tables

**Table 1:** Statistics for each glider mission and median over all gliders.

	**Name**	**Time**	**Length (days)**	**Profiles number**	**Depth max (m)**	**Offset QC CHL (mg m**^**−3**^)		**Temperature (*****°*****C)**		**Salinity**		**Density (kg m**^**−3**^)		**DO (ml l**^**−1**^)		**CHL (mg m**^**−3**^)	
		**start**	**end**					**mean**	**std**	**mean**	**std**	**mean**	**std**	**mean**	**std**	**mean**	**std**
1	Nemo 1	2008-11-25	2008-11-27	2.8	182	133	0.07 (189 m)	18.7	1.9	35.53	0.086	1025.7	0.5	4.3	0.5	0.6	0.5
2	Nemo 2	2009-03-17	2009-04-09	23	400	189	0.05 (191 m)	17.7	2.6	35.35	0.314	1025.8	0.9	3.8	0.5	0.3	0.4
3	Nemo 3	2009-10-02	2009-10-02	0.9	47	166	0.09 (207 m)	16.9	1.1	35.44	0.075	1026.1	0.4	4.4	0.4	0.5	0.4
4	Nemo 4	2009-10-28	2009-11-13	16.2	715	196	0.1 (179 m)	17.2	2.2	35.33	0.089	1026	0.7	4.4	0.7	0.3	0.4
5	Nemo 5	2010-03-09	2010-03-10	1.5	71	169	0.05 (201 m)	16.5	3.2	35.31	0.1	1026.1	0.9	3.6	0.5	0.6	0.8
6	Dory 2	2010-08-09	2010-08-14	5.6	115	104	0.02 (911 m)	19.7	0.8	35.51	0.03	1025.5	0.4	/	/	0.3	0.2
7	Nemo 6	2010-09-21	2010-10-06	15.2	737	179	0.06 (200 m)	16.5	1.4	35.45	0.09	1026.2	0.4	4.3	0.5	0.5	0.5
8	Nemo 7	2010-11-26	2010-12-15	19.5	2580	96	0.37 (98 m)	18.6	2.5	35.31	0.24	1025.5	0.7	4.1	1	0.6	0.8
9	Nemo 8	2011-05-19	2011-06-07	19	1584	94	0.16 (94 m)	22	0.5	35.44	0.05	1024.7	0.2	4.3	0.2	0.7	0.2
10	Nemo 9	2011-08-10	2011-08-31	21.9	1757	189	0.08 (197 m)	18	1.5	35.49	0.08	1025.8	0.4	4.4	0.3	0.4	0.3
11	Nemo 10	2011-11-12	2011-11-28	16.5	1752	111	0.13 (111 m)	18.7	1.7	35.46	0.09	1025.6	0.5	4.1	0.6	1.8	1.8
12	Nemo 12	2012-06-20	2012-07-11	21.5	2866	86	0.122 (86 m)	19.7	1	35.38	0.07	1025.2	0.3	4.4	0.3	0.5	0.3
13	Nemo 13	2012-07-15	2012-08-05	20.7	2135	93	0.14 (93 m)	19.7	0.7	35.45	0.03	1025.3	0.2	4.5	0.5	0.7	0.5
14	Nemo 14	2012-09-04	2012-09-25	21.6	1993	108	0.1 (108 m)	17.4	1.1	35.45	0.05	1025.9	0.3	4.2	0.6	0.7	0.7
15	Nemo 15	2012-11-14	2012-11-25	10.9	1259	87	0.27 (87 m)	21.2	1.5	35.43	0.05	1024.9	0.5	4	0.5	1.3	0.7
16	Dory 5	2013-05-31	2013-06-14	14.8	60	174	0.04 (1000 m)	18.8	2.7	35.41	0.08	1025.7	0.8	4.3	0.5	0.2	0.2
17	Nemo 16	2013-05-31	2013-06-20	20.5	1796	100	0.20 (100 m)	21.8	0.7	35.35	0.06	1024.7	0.2	4.2	0.2	0.3	0.2
18	Nemo 17	2013-10-09	2013-10-31	21.6	1868	106	0.71 (99 m)	19	1.8	35.48	0.09	1025.5	0.5	/	/	0.3	0.3
19	Nemo 18	2013-11-21	2013-12-12	20.8	2336	97	0.16 (100 m)	20.6	1.9	35.48	0.06	1025.1	0.6	4	0.6	0.9	0.7
20	Nemo 19	2014-03-25	2014-04-11	17.5	212	177	0.07 (208 m)	20.4	3	35.42	0.09	1025.1	0.9	3.5	0.5	0.8	0.7
21	Nemo 20	2014-06-24	2014-07-12	17.5	1010	194	0.07 (198 m)	20.1	1.4	35.48	0.04	1025.3	0.5	3.9	0.3	0.7	0.6
22	Nemo 21	2014-09-15	2014-10-09	24.1	1332	200	0.06 (200 m)	19.7	1.2	35.51	0.04	1025.4	0.4	4	0.5	0.6	0.4
23	Nemo 22	2015-02-11	2015-02-25	14.6	751	137	0.09 (137 m)	18.7	4.3	35.35	0.13	1025.5	1.1	/	/	0.9	1.5
24	Nemo 23	2015-04-17	2015-04-30	12.5	747	175	0.51 (196 m)	22	2.1	35.3	0.67	1024.6	0.8	4.7	0.6	0.3	0.4
25	Nemo 24	2015-06-02	2015-06-22	20.5	2241	195	0.01 (195 m)	21	1.4	35.60	0.1	1025.1	0.4	4	0.2	0.6	0.2
26	Nemo 25	2015-10-10	2015-12-02	22.7	2008	147	/	17.8	2.3	35.44	0.09	1025.8	0.6	3.9	0.6	/	/
	MEDIAN:			18.25	1295	142	0.09 (189 m)	18.9	1.6	35.44	0.08	1025.5	0.5	4.2	0.5	0.6	0.4
Columns show each name, start and end time (year-month-day), length of the mission, number of profiles on the shelf, maximum depth of profiles, offset used for the quality control of CHL (and deepest depth used)(see section 2.4 for more information), mean and s.d. of temperature, salinity, density, DO and CHL. (Note that highest CHL means for Nemo 10 were obtained from optical sensors factory calibrated 1 month before deployment. All calibration dates are available in the file’s metadata.)																	

**Table 2:** Variables in both NetCDF data files (‘ALL’ refers to common variables): name, dimension, description and units.

**File**	**Name**	**Dimension**	**Quick description**	**Units**
**ALL**	LATITUDE	I×J	Grid point location in latitude	degrees North
	LONGITUDE	I×J	Grid point location in longitude	degrees East
	DEPTH	DEPTH	Depth related to sea surface	m
	GLIDER	GLIDER	Glider missions, names, start and end dates, original file and deployment names, sensor types, serial numbers, calibration dates	
**Mean_hydrography_NSWshelf_gliders_2008-2015.nc**	Nb_missions	I×J×DEPTH	Number of glider missions	
	Nb_profiles	I×J	Number of profiles	
	TEMP	I×J×DEPTH	Mean seawater temperature	*°*C
	PSAL	I×J×DEPTH	Mean seawater salinity	
	DENS	I×J×DEPTH	Mean seawater density	kg m^−3^
	CPHL	I×J×DEPTH	Mean seawater chlorophyll-a fluorescence	mg m^−3^
	DOXY	I×J×DEPTH	Mean seawater dissolved oxygen	ml l^−1^
**Mean_hydrography_NSWshelf_per_glider_2008-2015.nc**	GLIDER_START_TIME	GLIDER	Starting date for each glider mission	Days since 1950-01-01
	GLIDER_END_TIME	GLIDER	End date for each glider mission	Days since 1950-01-01
	Nb_profiles	I×J×GLIDER	Number of profiles	
	TEMP	I×J× DEPTH×GLIDER	Mean seawater temperature	*°*C
	PSAL	I×J×DEPTH×GLIDER	Mean seawater salinity	
	DENS	I×J×DEPTH×GLIDER	Mean seawater density	kg m^−3^
	CPHL	I×J×DEPTH×GLIDER	Mean seawater chlorophyll-a fluorescence	mg m^−3^
	DOXY	I×J×DEPTH×GLIDER	Mean seawater dissolved oxygen	ml l^−1^
